# Validation of *Monilinia fructicola* Putative Effector Genes in Different Host Peach (*Prunus persica*) Cultivars and Defense Response Investigation

**DOI:** 10.3390/jof11010039

**Published:** 2025-01-06

**Authors:** Lucia Landi, Annamaria Lucrezia D’Ortenzio, Sarah Mojela Makau, Rita Milvia De Miccolis Angelini, Gianfranco Romanazzi

**Affiliations:** 1 Department of Agricultural, Food and Environmental Sciences, Marche Polytechnic University, Via Brecce Bianche, 60131 Ancona, Italy; a.l.dortenzio@staff.univpm.it (A.L.D.); u13322789@tuks.co.za (S.M.M.); g.romanazzi@univpm.it (G.R.); 2 Department of Plant and Soil Sciences, University of Pretoria, Private Bag X20, Hatfield, Pretoria 0028, South Africa; 3 Department of Soil, Plant and Food Sciences, University of Bari Aldo Moro, 70126 Bari, Italy; ritamilvia.demiccolisangelini@uniba.it

**Keywords:** brown rot, defense genes, fungal effector proteins, gene expression, stone fruit

## Abstract

*Monilinia fructicola* is the most common and destructive brown rot agent on peaches. Knowledge of gene expression mediating host–pathogen interaction is essential to manage fungal plant diseases. *M. fructicola* putative virulence factors have been predicted by genome investigations. The pathogen interaction with the host was validated. Five *M. fructicola* isolates were inoculated on two cultivars (cv.s) of peach (*Prunus persica* (L.) Batsch) ‘Royal Summer’ and ‘Messapia’ with intermediate and late ripening periods, respectively. The expression pattern of 17 candidate effector genes of *M. fructicola* with functions linked to host invasion and fungal life, and seven peach genes involved in the immune defense system were monitored at 0, 2, 6, 10, and 24 h-post inoculation (hpi). All fungal isolates induced similar brown rot lesions on both cv.s whereas the modulation of effector genes was regulated mainly at 2, 6, and 10 hpi, when disease symptoms appeared on the fruit surface, confirming the involvement of effector genes in the early infection stage. Although differences were observed among the fungal isolates, the principal component investigation identified the main differences linked to the host genotype. The salicylic acid and jasmonate/ethylene signaling pathways were differently modulated in the host independent from the fungal isolate used for inoculation. On plants susceptible to brown rot, the pathogen may have adapted to the host’s physiology by modulating its effectors as weapons.

## 1. Introduction

Brown rot is one of the most destructive diseases of stone and pome fruits (peach, nectarine, apricot, cherry, and plum) both in the field and postharvest [1]. The most important causal agents are the fungal species *Monilinia fructicola* (G. Winter) Honey, *Monilinia laxa* (Aderhold and Ruhland) Honey, and *Monilinia fructigena* (Aderhold and Ruhland) Honey. In the field, they induce indistinguishable symptoms on blossoms, twigs, branches, and fruits of susceptible host plants [2]. Estimates of losses are usually about 30% during the growing season [3]. Stone fruits with latent *Monilinia* infection develop disease symptoms at the market stage, leading to losses during postharvest storage that can exceed 80% [4,5]. Of the three closely related fungal species causing brown rot, *M. fructicola* is the most common on stone fruits and frequently found in North America, Australia, New Zealand, Japan, Brazil, and other South American countries [6]. In 2001, *M. fructicola* was reported in France on peach [7] and has rapidly spread to other European countries, becoming more prevalent than the former indigenous species [8,9,10]. Preharvest and postharvest brown rot disease management is performed using synthetic fungicide application in the orchard during the growing season [11]. The use of sustainable and environmentally friendly alternatives has increased in the last few decades [4,12,13]. However, for fruits such as peaches and nectarines, included in the list of the most produced fruits in Europe (FAOSTAT, 2022 http://www.fao.org/faostat/en/#data/QC, (accessed on 2 October 2024), brown rot is still highly destructive from fruit formation to storage [4].

The most commercial peach cultivars are susceptible to the infection. However, different susceptibility or moderately resistant cultivars to *M. fructicola* were reported [14,15,16,17]. Investigating the molecular mechanisms of plant–microbe interaction, fungal pathogenicity, and plant resistance is crucial to developing novel and safer strategies for the effective control of plant diseases. Plants have developed multilayered defense mechanisms finely tuned and complex to effectively prevent pathogen infections provoking an array of plant defense responses [18,19].

Several studies recently investigated the peach–*Monilina fructicola* interaction to underline the genetic basis of brown rot resistance. The proteomic investigation among peach cultivar differently susceptible to *M. fructicola* indicated that pathogen-affected proteins in peach cultivar moderately resistant to *M. fructicola* were mainly involved in energy and metabolism, while cultivar more susceptible accumulated proteins involved in disease/defense and metabolism [15]. Recently, several genes associated with brown rot resistance involved in hypersensitive response (HR), as well as genes related to lignin biosynthesis, calcium signaling, and autophagy, were suggested [17,20].

However, to defeat plant responses, many pathogens deploy a variety of effector proteins. Fungal effector proteins can be generally grouped based on their mode of deployment within the host effectors secreted into the apoplast or xylem of the host plant and cytoplasmic effectors translocated into host cells [21]. Effector proteins increase host susceptibility via multiple pathways, especially by suppressing plant defense responses [22]. Therefore, pathogen effector identification is critical for the development of crop disease resistance [23]. An improved understanding of fungal effector function and the associated underlying mechanisms, in conjunction with the use of host-resistant genotypes and induction of gene-silencing plants to obtain disease resistance, could be a critical strategy to prevent and control plant diseases in the future.

Genomics has revolutionized our ability to characterize pathogens, increasing public databases with new genomic resources. High-quality draft genomes of *M. fructicola* and other *Monilinia* species have recently become available [24,25,26,27,28,29]. Furthermore, they represent useful sources for investigations into the evolutionary history of the *Monilinia* genus within the *Sclerotiniaceae* family, as well as into pathogenicity mechanisms and host–pathogen interactions. The necrotrophic lifestyle associated with *Monilinia* spp. typically involves secretion of cell wall-degrading enzymes and toxic metabolites to destroy tissues, degrade plant cell wall components, and start infection [29,30,31,32]. However, more research is required to validate key virulence factors in the host-compatible responses.

With the aim to validate the in vivo involvement of putative effector genes of *M. fructicola* selected by genomic investigation [32], five different *M. fructicola* fungal isolates were inoculated in two host peach cv.s (*Prunus persica*), ‘Royal Summer’ and ‘Messapia’ with intermediate and very late ripening time maturation, respectively. After inoculation, the expression profiles of 17 putative effector genes and seven defense genes induced in peach fruits by *M. fructicola* colonization were analyzed at 0, 2, 6, 10, and 24 h-post inoculation (hpi).

## 2. Materials and Methods

### 2.1. Tested Fungal Isolates

The tested fungi were the monoconidic strains of *M. fructicola* AN7 and AN26 isolated from nectarine commercial orchards located in Fermo (Marche Region of Italy), and AN12, AN13, and AN14 isolated from peach commercial orchards located in Pesaro (Marche Region). The *M. fructicola* isolates were grown on Potato Dextrose Agar (PDA) medium at 25 °C and identified by polymerase chain reaction (PCR) using protocols described by Côte et al. [33]. For conidia preparation, 10 mL sterile water was added to the one-week-old PDA cultures. Conidia were collected by scraping the plates followed by filtration to remove mycelium and then resuspended in distilled water to a concentration of 10^9^ conidia/mL.

### 2.2. Virulence In Vivo Assay

The test was performed on two peach cv.s, ‘Royal Summer’ and ‘Messapia’, both susceptible to brown rot. The cv. ‘Royal Summer’ is characterized by early blossoming and intermediate ripening time maturation, while the cv. ‘Messapia’ is characterized by medium-late blossoming and very late ripening time. Fruits used for inoculation were collected in conventional orchards in the Marche region (Central-Eastern Italy) in 2022 at the ripening in mid-July for the cv. ‘Royal Summer’ and mid-September for the cv. ‘Messapia’. Fruits were surface sterilized in 1% sodium hypochlorite solution for 2 min, rinsed in sterile distilled water three times, and air-dried at room temperature. Then, four wounds (5 × 5 mm, deep × wide) were made with a sterile nail around the equator of peach fruits. Artificial infection was performed by inoculating 30 μL of conidia suspension (10^9^ conidia/mL) in each wound. The control fruits were inoculated with 30 μL sterile distilled water. For each treatment, three peach fruits were inoculated and kept in the dark at a temperature of 25 °C and relative humidity of 90% for a maximum of 24 h. The inoculated peach sample was collected at 0, 2, 6, 10, and 24 hpi. The five sampling time points included an initial asymptomatic invasion stage and a necrotrophic life stage with brown rot lesion and evident pathogen sporulation. After the lesion diameter was measured at each inoculation point [10], sample material was collected from each lesion, frozen with liquid N immediately after sampling, and stored at −80 °C until RNA extraction. After inoculation, the rest of the conidia suspensions from each *M. fructicola* isolate were stored at −80 °C until RNA extraction and used as positive control.

### 2.3. Gene Expression Investigation

The genes analyzed in this work were 17 putative pathogenic effector genes identified by EffectorP 1.0, EffectorP 2.0, and EffectorP 3.0 https://effectorp.csiro.au (accessed on 11 March 2024) prediction tools in *M. fructicola* genome with or without orthologs with *M. laxa*, *M. fructigena*, *Botrytis cinerea*, and *Sclerotinia sclerotiorum* [32] (Table 1), and seven key genes linked to plant defense in peach (Table 2). The gene expression analysis was carried out by reverse transcription-quantitative real-time PCR (RT-qPCR) using a SYBR-green dye system, according to Minimum Information for Publication of Quantitative Real-Time PCR Experiments (MIQE) guidelines [34].

#### 2.3.1. RNA Extraction

RNA from both peach and fungal mycelium was extracted following the methodology of Molina Hernandez et al. [35]. Briefly, 1 mL of the CTAB extraction buffer [36] was added to samples. The efficient disruption of biological material in each sample was then obtained using the TissueLyser III (Qiagen, Hilden, Germany) at 30 Hz for 30 s, repeated twice. Following incubation at 65 °C for 40 min, the supernatant was recovered and vigorously mixed with an equal volume solution of chloroform/isoamyl alcohol (24:1) for 15 s and finally centrifuged at 10,000× *g* for 8 min at 4 °C. This last step was repeated twice. The total RNA was precipitated overnight at 4 °C, adding 0.25 µL 10 M LiCl. Finally, samples were washed with 70% ethanol, and the pellet obtained was resuspended in 50 µL of Milli-Q water. The quantity and quality of the RNA extracted was assessed based on an absorbance ratio of 1.80 to 1.90 at 260/280 nm and 1.8 to 2.0 at 230/260 nm, using BioPhotometer Plus (Eppendorf Inc., Westbury, NY, USA). For each sample, two RNA extractions were performed. Then RNA solutions were pooled before the next step.

#### 2.3.2. Reverse Transcription

For each sample, 0.5 ng of RNA was used for cDNA synthesis, performed using iScript TM cDNA synthesis kits (Bio-Rad Laboratories, Hercules, CA, USA) according to the manufacturer’s instructions. For each sample, the products from cDNA synthesis were performed twice to obtain an adequate quantity of cDNA to analyze the different genes. The mixed products obtained (40 µL each) were diluted (1/10) according to preliminary assessments.

#### 2.3.3. Primers and Reference Gene Selection

Target and reference genes were chosen from the gene sequences of *M. fructicola* (NCBI GenBank accession number GCA_008692225.1) and peach (*P. persica*) (GenBank accession number GCF_000346465.2 deposited in NCBI GenBank). The accession code and main function of target genes are reported in Table 1. Concerning the reference genes used for gene expression normalization, the β-tubulin cofactor d protein (EYC84_002797), β-actin (EYC84_004375), and elongation factor 1-alpha (EYC84_000787) were selected for *M. fructicola*, while β actin (XM_007205304.2), tubulin beta-1 chain (XM_007217941.2), EF-1 elongation factor 1-alpha (XR_002271124.1), and histone H1 (XM_034347152.1) were selected for peach fruits. The primer sets were selected using the Primer3 software (version 0.4.0) https://bioinfo.ut.ee/primer3-0.4.0 (accessed on 8 April 2024). The primer pairs were chosen and validated in silico using primer BLAST-specific analysis (https://blast.ncbi.nlm.nih.gov/Blast.cgi accessed on 30 December 2024) and then from RT-qPCR, as described later, according to the melting profiles obtained from cDNA extracted from uninoculated peach cv.s ‘Messapia’ and ‘Royal Summer’, and *M. fructicola* conidia. The stabilities of candidate reference genes were evaluated using algorithms geNorm module of qbase + (Biogazelle) [37]. These algorithms rank the reference genes based on the stability value (M-value). A lower M-value corresponds to a more stable gene. The recommended stability for homogenous samples is M-value < 0.5 (coefficient of variation, (CV) < 0.25), and for heterogeneous samples, it is M-value < 1 (CV < 0.5) [34].

**Table 1:** Target genes selected for gene expression analysis in *M. fructicola*. The main functions of the gene products are also given.

LOCUS TAG	Code	Name	Function
^a^ EYC84_006718	NEP1	necrosis- and ethylene-inducing protein 1	This family consists of several NePs, including necrosis- and ethylene-inducing proteins from oomycetes, fungi, and bacteria [38,39].
^b^ EYC84_009186	NEP2	necrosis- and ethylene-inducing protein 2	
^b^ EYC84_007944	Egh16	Egh16-like virulence factor	Egh16-like virulence factors are found in many pathogenic filamentous fungi and are thought to play a role in host interaction [40].
^b^ EYC84_002620	CVNH	CVNH—CyanoVirin-N homology	CyanoVirin-N homology domains are found in the sugar-binding antiviral protein cyanovirin-N (CVN) and filamentous ascomycetes. In *S. sclerotiorum*, CVNH was associated with plant infection and was proposed to suppress plant resistance hypersensitive response [41].
^b^ EYC84_008853	PG6	polygalacturonase 6	Polygalacturonase (PG) is one of the main enzymes in fungal pathogens to degrade plant cell walls, facilitating penetration and colonization of the host [42].
^b^ EYC84_010609	PME	pectin methylesterase and related acyl-CoA thioesterases	Pectin methylesterase is the first enzyme acting to modify pectins in plant cell walls [43].
EYC84_008964	GAS1	probable GAS1 Glycophospholipid-anchored surface glycoprotein	GAS1 elongates the β-1,3-glucan chains and plays an important role in the biosynthesis of the fungal cell walls. GAS1 induces typical hypersensitive response and systemic acquired resistance (SAR) defense responses [44]
^d^ EYC84_001420	HsbA	hydrophobic surface-binding protein A	HsbA is a specific fungal spore-secreted protein that can bind to hydrophobic surfaces. These proteins can recruit lytic enzymes to the surface and promote their degradation [45].
^b^ EYC84_008014	SSP	small secreted protein	Comprising 40–60% of the total fungal secretome, SSPs are defined as proteins that contain a signal peptide and a sequence of less than 300 amino acids [46].
^a^ EYC84_003936	GELP	GDSL-type esterase/lipase proteins (GELPs)	GELPs represent a variety of lipolytic enzymes that hydrolyze diverse lipidic substrates, including thioesters, aryl esters, and phospholipids [47]
^c^ EYC84_000899	TLP	osmotin/thaumatin-like superfamily	TLPs belong to the pathogenesis-related 5 protein family in plants, playing a significant role in host defense [48]. In fungi, the secretion activity and endo-β-1,3-glucanase activity of the TLP family members were detected [49].
^c^ EYC84_005201	Rnt2	ribonuclease T2-like	The Rnt2 family was identified during the plant infection and contributes to the virulence of the pathogen through the degradation of plant RNA [50].
^b^ EYC84_002145	ycaC	isochorismatase	Isochorismatases involved in the cellular amino acid catabolic process have been found in the secretome of phytopathogens. They are a precursor of salicylic acid and many other distinct derivatives in plants, fungi, and bacteria [51].
^a^ EYC84_004547	RPC5	RPC5 protein	This family represents the RPC5 protein, which is part of the RNA polymerase III complex. It acts as a nuclear and cytosolic DNA sensor involved in innate immune response and can sense non-self dsDNA that serves as a template for transcription into dsRNA [52].
^a^ EYC84_003395	UkE1	hypothetical protein	Unknown
^b^ EYC84_005228	UkE2	hypothetical protein	
^e^ EYC84_011230	UkE3	hypothetical protein	

Note: orthologs with: ^a^ = M. laxa, M. fructigena; ^b^ = M. laxa, M. fructigena, Botrytis cinerea, and Sclerotinia sclerotiorum; ^c^ = M. fructigena, Botrytis cinerea, and Sclerotinia sclerotiorum; ^d^ = M. laxa, M. fructigena, and Botrytis cinerea; ^e^ = M. laxa, Sclerotinia sclerotiorum, and Botrytis cinerea.

**Table 2:** Target genes selected for gene expression analysis in *P. persica.* The main functions of the gene products are also given.

Locus Tag	Code	Name	Function
NM_001405051.1	PG	polygalacturonase	PGs are an important pectolytic glycanase, primarily implicated in the softening of fruit during ripening [53].
AF124527.1	ETR-1	ethylene receptor, transcript ETR1	ETR-1 is included in the ethylene receptor family [54].
XM_007203058.2	SA	salicylic acid-binding protein 2	This protein is required to convert methyl salicylate to salicylic acid as part of the signal transduction pathways that activate SAR in systemic tissue [55].
JF694923.1	PR-1	pathogenesis-related protein 1	PR-1 constitutes a secretory peptide family. This protein plays important roles in plant metabolism in response to biotic and abiotic stresses, and its role in plant defense has been widely demonstrated [56].
XM_020560994.1	JA	jasmonic acid-amido synthetase	JA is involved in jasmonic acid defense biosynthesis [57].
XM_007215636.2	ACC	1-aminocyclopropane-1-carboxylate oxidase	ACC catalyzes the final step in ethylene biosynthesis [58].
XM_007215891.2	GST	glutathione S-transferase F12	GST has been implemented in diverse plant functions, such as detoxification of xenobiotic, secondary metabolism, growth and development, and especially against biotic and abiotic stresses [59].

#### 2.3.4. Quantitative Real-Time PCR

The RT-qPCR reactions were carried out in duplicate in a total volume of 10 µL each, which contained 4.6 µL of diluted cDNA, 0.10 µM of each primer, and 5 µL of 2 × SsoAdvanced Universal SYBR Green Supermix, in CFX Connect thermal cycle (CFX Connect, Bio-Rad Laboratories). The PCR cycling conditions were 95 °C for 3 min, followed by 40 cycles of 95 °C for 15 s, and 60 °C for 45 s. Melting curve analysis was performed over the range of 68–98 °C. The assays included cDNA from *M. fructicola* fungal isolates, uninoculated peach fruits, and no-template controls to determine the nonspecific amplification. The RT-qPCR efficiency (E) of each primer pair was determined using standard curves generated according to E = 10 − 1/slope. In detail, for the standard curve, the diluted cDNAs from samples (10 μL each) were mixed, and then four serial dilutions 1:5 (initial dilution, 0.2, 0.04, 0.008) were obtained. The comparative 2^(−ΔΔCt) method [60] was used for the analyses. The 0 hpi was used as the control sample for both effector and peach gene expressions.

### 2.4. Statistical Analysis

For each isolate, three peach fruits were inoculated, and the experiment was repeated twice. All the data were expressed as means ± standard deviation and subjected to one-way ANOVA with Tukey HSD post hoc test. Differences were considered statistically significant for *p* < 0.05. To explain a large fraction of the observed variability among different gene expressions of effectors in *M. fructicola* isolates after peach inoculation, principal component analysis (PCA) was performed and computed using the PCA function (FactoMineR) (package R_2.9.tar.gz) [61]. Then, the factoextra R package (1.0.7.tar.gz) was used to produce ggplot2-based visualization of the PCA results [62].

## 3. Results

### 3.1. In Vivo Test: Virulence Assay

**Figure 1:**
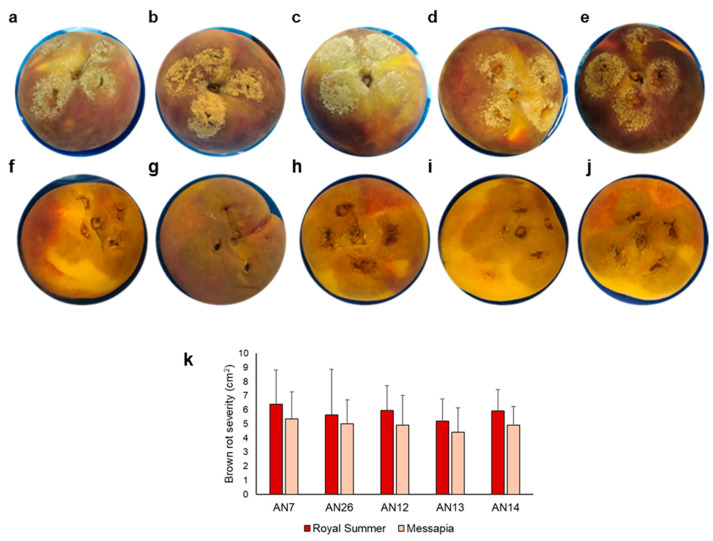
Disease assessment on wounded peach fruit cv. ‘Royal Summer’ (**a**–**e**) and cv. ‘Messapia’ (**f**–**j**) 24 h post inoculation (hpi) with 30 μL conidia suspension (10^9^ conidia/mL) of AN7 (**a**,**f**), AN26 (**b**,**g**), AN12 (**c**,**h**) AN13 (**d**,**i**), and AN14 (**e**,**j**) *M. fructicola* isolates. Brown rot severity (**k**) caused by *M. fructicola* isolates at 24 hpi after inoculation on peach fruit cv. ‘Royal Summer’ and cv. ‘Messapia’. Data represent the mean of two experiments with four inoculations per fruit and three fruits per isolate and experiment (n = 24). Data were analyzed by analysis of variance. Means and standard deviation are not significantly different (*p* ≤ 0.05) according to the Tukey HSD post hoc test.

Virulence of *M. fructicola* isolates AN7, AN26, AN12, AN13, and AN14 were compared. No significant differences were observed regarding brown rot severity related to lesion diameter among inoculated isolates on both cv.s ‘Royal Summer’ and ‘Messapia’ (Figure 1). However, differences were observed in fungal sporulation on the two cultivars. At 24 hpi, all isolates showed evident fungal sporulation on ‘Royal Summer’ surface while sporulation was rarely present on ‘Messapia’ (Figure 1).

### 3.2. Gene Expression Analysis

Specific primer sets were selected for both putative effector and peach genes analyzed (Supplementary Material Table S1). The melting curve analyses for all the amplicons showed a single peak, and no specific products or primer–dimer formation was detected with the amplification of the samples. The standard curves generated for each gene showed high efficiency of each specific primer pair for RT-qPCR ranging from 92.2 to 108.3% (Supplementary Table S1). The specificity of the primers was confirmed by analyzing cDNA from uninoculated peaches or *M. fructicola* isolates.

In the gene expression effector investigation, among the three putative candidate reference genes, the lowest M-values were detected for the β-tubulin. This indicated that these two reference genes were suitable for the RT-qPCR investigation. Concerning the four candidate reference genes tested on peach, the most suitable reference genes were β-actin and histone H1 (Table 3).

The gene expression data are discussed according to their relative fold-changes compared to relevant controls.

#### 3.2.1. Expression of *M. fructicola* Putative Effector Genes

**Figure 2:**
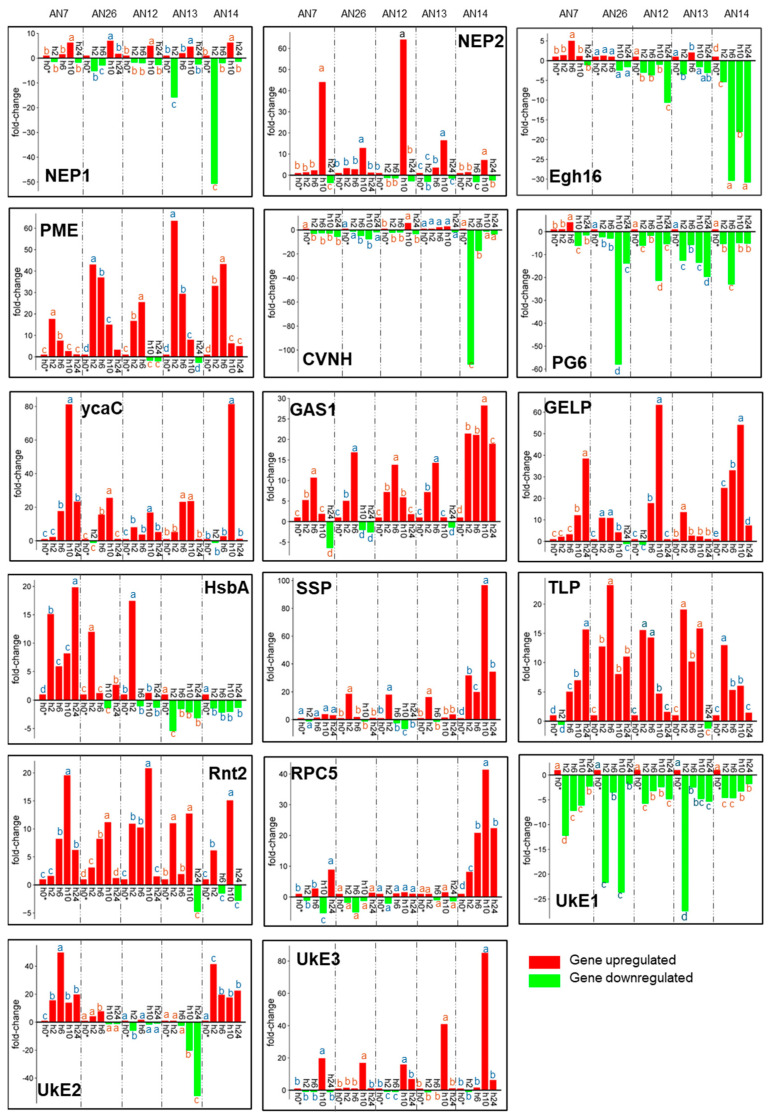
Expression profiles of 17 putative effector genes assayed (Table 1) by RT-qPCR. RNA was isolated from five *M. fructicola* isolates (AN7, AN26, AN12, AN13, and AN14) after inoculation on peach cv. ‘Royal Summer’ at 0, 2, 6, 10, and 24 hpi. The data were represented as fold-change compared to 0 hpi (h0*), which is given a value of 1. Means were determined with data from two biological replicates with three technical replicates each (n = 6). For each gene of each fungal isolate, columns with different letters are significantly different (*p* ≤ 0.05; Tukey HSD post hoc test).

When *M. fructicola* was inoculated on the cv. ‘Royal Summer’, the expression of effector genes changed according to both sampling time and fungal isolates. Overall, the fold-change values of putative effector genes of *M. fructicola* inoculated on ‘Royal Summer’ ranged from -112-fold (CyanoVirin-N, CVNH, gene, isolate AN14 at 2 hpi) to 97-fold (Small Secreted Protein (SSP) isolate AN14 at 10 hpi) (Figure 2).

The necrosis- and ethylene-inducing protein 1 precursor (NEP1) and necrosis- and ethylene-inducing protein 2 precursor (NEP2) genes modulated their expression over time. Interestingly, both were significantly upregulated only at 10 hpi (Figure 2). Other genes, including ribonuclease T2-like (Rnt2), isochorismatase/cysteine hydrolases, (ycaC) and hypothetical protein 3 (UkE3), showed maximum expression level at 10 hpi but were upregulated also in different time points. Pectin methylesterase (PME), glycophospholipid-anchored surface glycoprotein (GAS1), GDSL-type esterase/lipase proteins (GELP), osmotin/thaumatin-like superfamily/PR-5 (TLP), and SSP were mainly upregulated over time reaching maximum peaks of expression at different hpi based on the isolates. The genes hydrophobic surface-binding protein A (HsbA), RPC5, and hypothetical protein 2 (UkE2) showed greater variability among isolates and over time. These genes are downregulated in some fungal isolates and upregulated in others. Finally, the Egh16-like virulence factor (Egh16), CVNH, polygalacturonase 6 (PG6), and hypothetical protein 1 (UkE1) showed a quick downregulation starting from 2 hpi, but large fold-change differences among isolates were observed (Figure 2).

**Figure 3:**
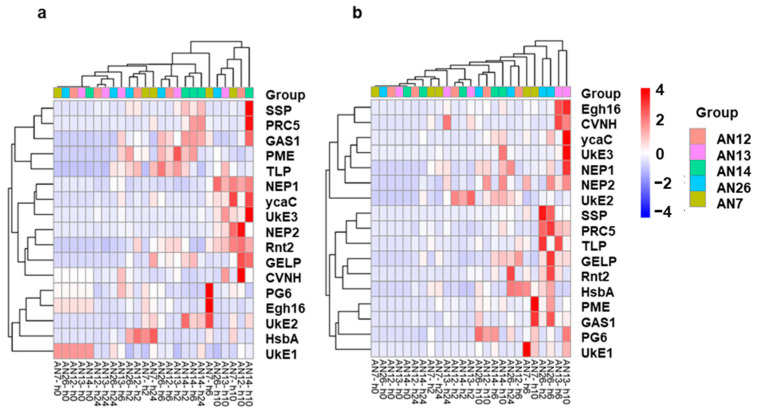
Heatmap showing hierarchical cluster analysis of 17 *M. fructicola* putative effector genes (Table 1) expressed across all samples (different fungal isolates AN7, AN26, AN12, AN13, and AN14 at 0, 2, 6, 10, and 24 hpi) on the two cv.s ‘Royal Summer’ (**a**) and ‘Messapia’ (**b**). The genes (rows) and samples (columns) are clustered using the Pearson Correlation distance and complete linkage hierarchical clustering. The gradient scale represents expression levels, with red showing the highest expression to blue with the lowest expression level.

The heatmap based on hierarchical clustering related to *M. fructicola* effector genes expressed when inoculated in the ‘Royal Summer’ cv. revealed that fungal isolate groups do not cluster together while specific expression trends of genes across the time were detected. The differentially expressed effector genes were clustered into three groups. Among the top genes across isolates, the Egh16, PG6, UkE2, HsbA, and UkE1 clustered together while another group was represented by SSP, RPC5, GAS1, PME, and TLP, and then finally NEP1, ycaC, UkE3, NEP2, Rnt2, GELP, and CVNH (Figure 3a).

**Figure 4:**
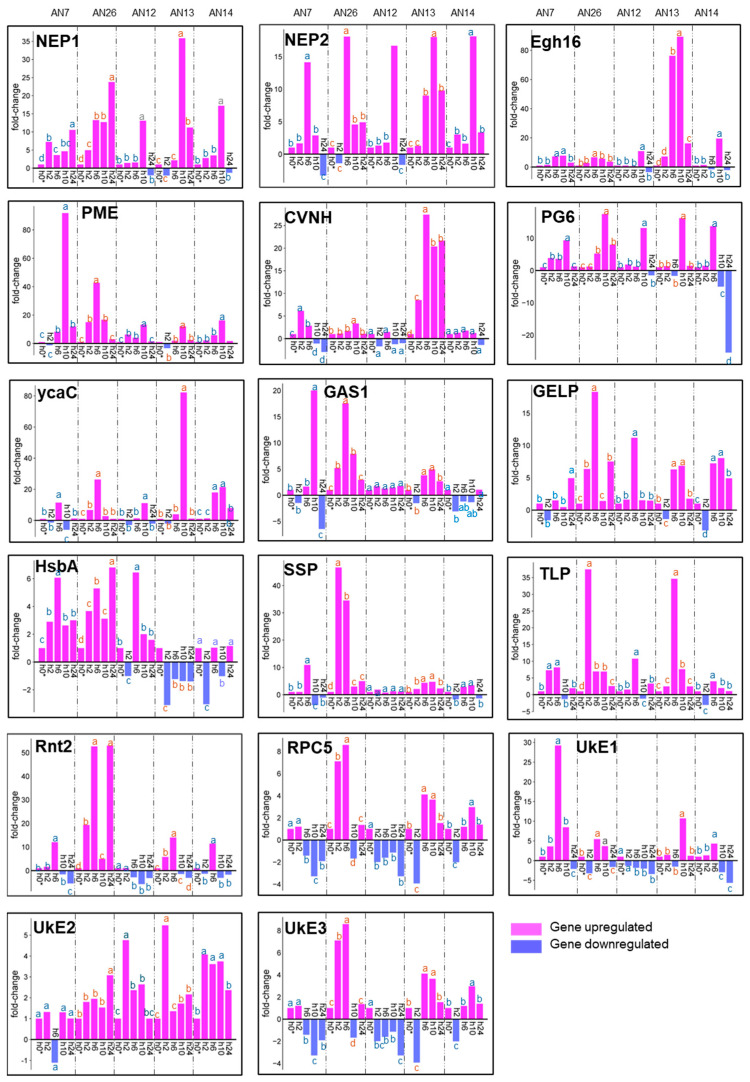
Expression profiles of 17 putative effector genes assayed (Table 1) by RT-qPCR. RNA was isolated from five *M. fructicola* isolates (AN7, AN26, AN12, AN13, and AN14) after inoculation on cv. ‘Messapia’ peach at 0, 2, 6, 10, and 24 hpi. The data were represented as fold-change compared to 0 hpi (h0*), which is given a value of 1. Means were determined with data from two biological replicates with three technical replicates each (n = 6). For each gene of each fungal isolate, columns with different letters are significantly different (*p* ≤ 0.05; Tukey HSD post hoc test).

When inoculated on the ‘Messapia’ cv., the gene expression of putative effector genes changed depending on both hpi and fungal isolates. Overall, the fold-change values ranging from −25-fold (PG6 AN26 isolate at 24 hpi) and 91-fold (PME, AN7 isolate at 10 hpi) were recorded (Figure 4). However, the genes NEP1, NEP2, Egh16, PME, CVNH, PG6, ycaC, GAS1, GELP, SSP, TLP, and UkE2 were primarily upregulated but with differences among isolates, related to fold-change values and time point of maximum expression peak. Finally, the genes HsbA, Rnt2, RPC5, UkE1, and UkE3 were also downregulated in several isolates (Figure 4).

The heatmap based on hierarchical clustering related to *M. fructicola* inoculated on ‘Messapia’ showed that the effector gene expression profile from fungal isolate groups was not distinct, while specific expression trends of genes across hpi were detected. The effector genes clustered into three main groups. One included Egh16, CVNH, ycaC, UkE3, NEP1, NEP2, and UKE2, another group was related to PME, GAS1, PG6, and UkE1, and at the last was SSP, RPC5, TLP, GELP, Rnt2, and HsbA, genes (Figure 3b).

#### 3.2.2. Expression of Peach Defense Genes

After the *M. fructicola* inoculation, the expression of several key genes involved in the major signaling pathways of plant defense mechanism showed differences between the peach cv.s, but with a similar trend and fold-change value according to the tested fungal isolates.

**Figure 5:**
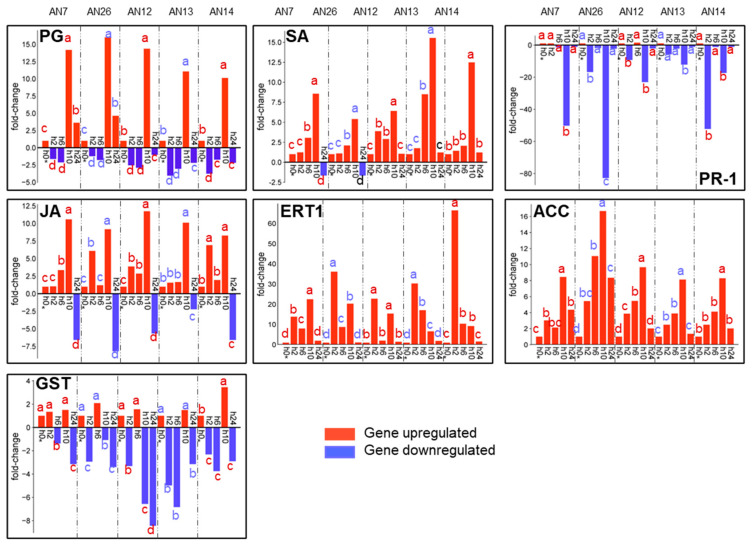
Expression profiles of seven key peach defense genes (Table 1) assayed by RT-qPCR detected in ‘Royal Summer’ after inoculation with AN7, AN26, AN12, AN13, and AN14 *M. fructicola* fungal isolates at 0, 2, 6, 10, and 24 hpi. The data were represented as fold-change compared to 0 hpi (h0*) which is given a value of 1. Means were determined with data from two biological replicates with three technical replicates each (n = 6). For each gene of each fungal isolate, columns with different letters are significantly different (*p* ≤ 0.05; Tukey HSD post hoc test).

Concerning the cv. ‘Royal Summer’ the polygalacturonase (PG), salicylic acid-binding protein 2 (SA), jasmonic acid-amido synthetase (JA), and 1-aminocyclopropane-1-carboxylate oxidase (ACC) were mainly overexpressed with a maximum expression peek at 10 hpi. The fold-change values at 10 hpi based on the different inoculated fungal isolates were from 11- to 16-fold for PG, from 6- to 12-fold for SA, from 8- to 11-fold for JA, and from 8.5- to 16.5-fold for ACC. The ethylene receptor transcript ETR1 (ERT1) gene was mainly upregulated at 2 and 10 hpi. Interesting PG gene expression was downregulated at 2 and 6 hpi for all inoculated isolates by 2- to 4-fold compared to control. Inversely, the gene expression of pathogenesis-related protein 1 (PR-1) was mainly downregulated at 10 hpi from −10 to −83-fold. The expression profile detected for glutathione S-transferase (GST) gene was more variable but was always downregulated at 24 hpi from −3 to −8.5-fold (Figure 5).

**Figure 6:**
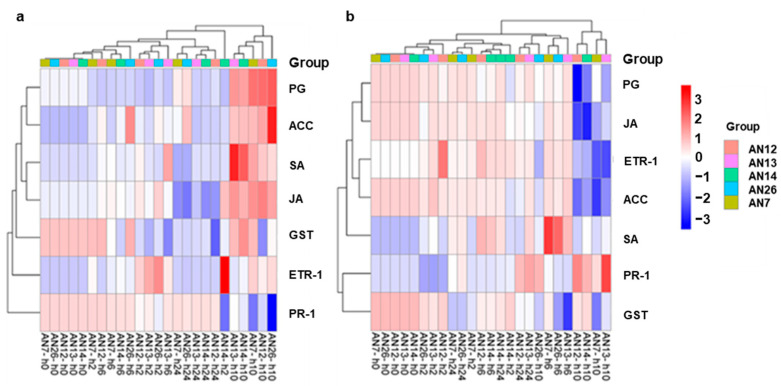
Heatmap showing hierarchical cluster analysis of seven genes (Table 1) expressed in peaches across all samples on the ‘Royal Summer’ cv. (**a**) and ‘Messapia’ cv. (**b**) after inoculation with AN7, AN26, AN12, AN13, and AN14 *M. fructicola* fungal isolates at 0, 2, 6, 10, and 24 hpi. The scale represents expression levels, with red showing the highest expression to blue with the lowest expression.

The heatmap based on a hierarchical cluster related to the ‘Royal Summer’ cv. inoculated with *M. fructicola* isolates revealed that gene expression profiles from fungal isolate groups were not distinct, while specific expression trends of genes across hpi were detected. The different peach defense genes were clustered in two groups: PG and ACC clustered together, and SA and JA together. The other genes all joined these two gene clusters (Figure 6a).

**Figure 7:**
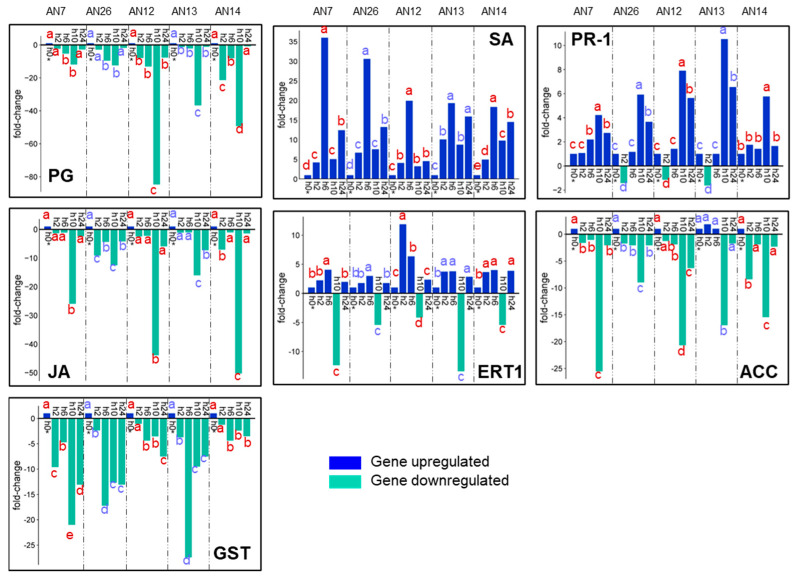
Expression profiles of seven key peach defense genes (Table 1) assayed by RT-qPCR detected in ‘Messapia’ after inoculation with *M. fructicola* fungal isolates AN7, AN26, AN12, AN13, and AN14 at 0, 2, 6, 10, and 24 hpi. The data were represented as fold-change compared to 0 hpi (h0*) which is given a value of 1. Means were determined with data from two biological replicates with three technical replicates each (n = 6). For each gene of each fungal isolate, columns with different letters are significantly different (*p* ≤ 0.05; Tukey HSD post hoc test).

A different pattern profile of gene expression was detected in the ‘Messapia’ cv. after *M. fructicola* inoculation compared to the ‘Royal Summer’ cv. The PG, JA, ACC, and GST were downregulated at the different time points and based on the different fungal isolates inoculated. The downregulation peak of PG was at 10 hpi in all peach samples regardless of the fungal inoculum tested (from 12- to 85-fold). Furthermore, the JA and ACC genes exhibited peak downregulation at 10 hpi from 12.5- to 50-fold and 9- to 25.5-fold, respectively. The GST gene was downregulated at all time points, especially at 6 hpi (27-fold after AN13 strain inoculation) and at 10 hpi (21-fold after AN7 inoculation). However, the SA and PR-1 genes were upregulated. The SA showed peak expression at 6 hpi (from 19- to 36-fold), and the PR-1 had a peak expression at 10 hpi (from 4- to 10.5-fold) (Figure 7).

The heatmap based on hierarchical cluster related to the ‘Messapia’ cv. inoculated with *M*. *fructicola* isolates revealed that gene expression profile from fungal isolate groups was not distinct; however, specific expression trends of genes across hpi were detected. The different peach defense genes were clustered in three groups: PG and JA clustered together, ETR1 and ACC joined by the SA gene, and the last group was related to PR-1 and GST (Figure 6b).

**Figure 8:**
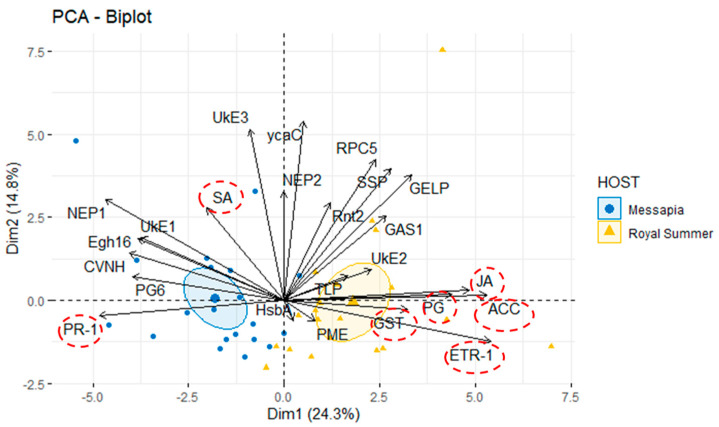
Principal component analysis (PCA) of gene expression dataset. PCA biplot (score plot) visualizing projections onto the first two principal components. The biplot graph represents the relationship among the expression of 17 putative effector genes (Table 1) and 7 peach expression genes highlighted by a red circle (see Table 1 for details) used as vectors and the peach cv.s ‘Messapia’ and ‘Royal Summer’ used as groups. The two main components (Dim1 and Dim2) explained 24.3% and 14.8% of the variance, respectively. Different colors and symbols represent the different cv.s: (• blue), ‘Messapia’ and (▲ yellow), ‘Royal Summer’. Ellipses indicate a confidence interval of 50%. The direction and length of the arrows illustrate how each effector (vector) contributes to the first two components in the PCA. Aligned vectors indicate a strong positive correlation between the two effectors (<30%). Vectors at right angles/opposites (>90%) indicate no correlation/negative correlation, respectively.

The dataset was analyzed using multivariate PCA to identify any structured variation contained in the dataset indicating an overall change of the gene expression pattern (i.e., gene expression data of 17 putative effector genes and 7 stress/defense genes for five *M. fructicola* isolates in two peach cv.s). The trait biplot analysis for the first two principal components, PC1 (Dim1) and PC2 (Dim2), accounted for 24.3% and 14.8% variability, respectively. Although no absolute distinct clustering was evident, limited overlap occurred in the gene expression according to the peach cv. based on the two orthogonal linear combinations of the features (Figure 8), while no separate clustering was observed in relation to the isolates or hpi. The NEP1, UkE1, Egh16, CVNH fungal effector genes, and PG6, as well as SA and PR-1 peach defense genes, contribute to the variability in gene expression detected in the ‘Messapia’ cv. host, while RPC5, SSP, GELP, Rint2, GS1, TLP, and UkE2 effector genes with JA, ACC, PG, ETR-1, and GST host defense genes presented the highest contribution in the ‘Royal Summer’ cv. host. These two groups were mostly developed on PC1 but seem to be uncorrelated with each other (the angle between these vectors is approximately >90°), while the NEP2, ycaC, and UkR3 putative effectors exhibited large positive loadings on PC2 and overlapped for the cv.s. The UkE2, TLP, HsbA, and PME closer to 0 indicate that the variable had a weak influence on the PC1 and PC2 components (Figure 8).

## 4. Discussion

Effector genes encoding molecules involved in disease establishment are expressed throughout the lifecycle of plant-pathogenic fungi. However, little is known about how effector gene expression is regulated.

In this work, we verified if some putative effector genes identified in the *M. fructicola* genome [32] were modulated in the peach host in the early infection phase. Then we performed tests based upon artificial fruit inoculation of five different *M. fructicola* fungal isolates on two peach cv.s showing differences in the ripening period, ‘Royal Summer’ and ‘Messapia’, with intermediate and very late ripening time, respectively. In addition, some key genes induced by *M. fructicola* linked to the first layer of the plant immune system were analyzed in peach fruits. We highlighted that all fungal isolates showed a similar ability to induce lesions associated with brown rot. Despite the similar lesion diameters, more abundant fungal sporulation was observed on the cv. ‘Royal Summer’ regardless of the isolates tested, compared to the cv. ‘Messapia’, suggesting differences between the two host peaches rather than between the fungal isolates. Currently, data are available showing a difference in susceptibility among these cv. to *Monilinia* spp. in the orchard. Previous experiments have also highlighted their susceptibility to *Monilinia* spp. (CRA Project 2009; MACFRUT2016). During the infection monitoring stage, all effector genes were differentially expressed over time, depending on the fungal isolates and largely on the hosts, which suggested specialized functions for these effector activities. The overall expression of the genes analyzed was regulated mainly at 2, 6, and 10 hpi when disease symptoms started to appear on the fruit surface, confirming the involvement of effector genes in the early infection stage [63]. In each peach cv., the gene expression differs among fungal isolates primarily on activation times and fold-change values. The PCA investigation highlighted the distinct clustering evident in the gene expressions, mainly according to the peach cv. Research indicated that each peach cv. has unique characteristics linked to nutrients, proteins, minerals, vitamins, and carbohydrates [64]. Therefore, although no differences were detected in the brown rot severity in the peach cv., physiological characteristics of the peaches may have contributed differentially in activating and modulating the expression of putative virulence effectors identified in the *M. fructicola* necrotrophic pathogen. In this regard, previous work established key differences in cultivars with different ripening periods involving a change of expression of the genes responsible for cell wall modification, redox signaling, hormonal regulation, and reception [65].

Based on these results, most effector genes linked to cv. ‘Royal Summer’ have heterogenous functions with roles in both the host invasion and fungal life. We refer to GELP involved in lipid metabolism [47], highly expressed during *B. cinerea* plant interactions [66], GAS1, which plays an important role in the biosynthesis of the fungal cell wall that can affect plant defense response [44], and TLP with secretory and endo-β-1,3-glucanase activity [49]. In addition, Rnt2 and RPC5 are involved in RNA synthesis. The first contributes to the virulence of pathogen through the degradation of plant RNA [50], while RPC5 codifies for a component DNA-directed RNA polymerase III affecting an innate immune response in plants by synthesizing small RNAs acting as a fungal pathogen effector that silences host target genes to promote infection, a virulence mechanism called cross-kingdom RNA interference (RNAi) [51,67].

On the other hand, the brown rot virulence factors that differentiate the cv. ‘Messapia’ from the cv. ‘Royal Summer’ act in the early infection stage of the host affecting necrosis [45]. These factors include PG6 involved in pectin degradation to help penetration and colonization of the host [42]; Egh16 gene found in many pathogenic filamentous fungi and thought to play a role during the early infection stage [40]; as well as the NEP1 and CVNH genes linked to necrosis induction in the host [68]. These genes were upregulated in the cv. ‘Messapia’ but were primarily downregulated in cv. ‘Royal Summer’. We hypothesize that the interaction with the host modulates their downregulation in the first 24 h, while other genes contributed to the onset of the disease in ‘Royal Summer’.

Our work indicates that the host can modulate the expression of virulence effector genes. Focusing our discussion on the NEP putative effector genes previously investigated in different plant–pathogen interactions, in the cv. ‘Royal Summer’, NEP1, and NEP2 genes were primarily upregulated only at 10 hpi, while in ‘Messapia’, they were upregulated at different times with the highest variability among *M. fructicola* isolates. Previous work evidenced a different contribution in the different phases of host infection of NEP-like proteins [69]. NEPs are necrosis- and ethylene-inducing proteins typically present in necrotrophic fungi, including *B. cinerea* [70,71] and *Sclerotinia sclerotiorum* [72]. NEPs, detected as virulence factors in several fungi, probably function by permeabilizing the plasma membrane [73,74]. NEP1 was found to interact with a single plasma membrane receptor involved in both mediating virulence and triggering defense [75]. Infiltration of NEP1 into leaves of *Arabidopsis thaliana* plants resulted in transcript accumulation of pathogenesis-related (PR) genes, production of ROS and ethylene, callose apposition, and HR-like cell death [76]. However, in *B. cinerea*, single knockout mutants in NEP1 and NEP2 coding genes using *Arabidopsis* did not affect virulence [77]. Furthermore, leaf infiltration of NEPs from *Botrytis squamosa* showed that onion cultivars are differentially sensitive to NEPs [39], while *M. fructicola* ortholog to NEP1 proteins from *Botrytis* spp. was able to induce cell death in *Nicotiana benthamiana, Solanum lycopersicum* (non-hosts), and *Prunus* spp. (natural hosts) leaves [31]. However, among the NEPs, only NEP2 upregulation was confirmed by transcriptomic investigation in different *P. persica* cv. after *M. fructicola* inoculation on fruits [78]. These examples suggest the complex nature of effector genes. In *M. fructicola*, the roles of effectors could be diverse, either in suppressing host immune responses during the early, biotrophic phase of the infection or inducing plant cell death in the host [29]. In our work, the investigation about key genes associated with the plant’s defense and stress mechanisms on peach highlighted a different response between the two cv.s but no link was found with the *M. fructicola* isolates inoculated. Despite the evident susceptibility to the pathogen, the host response was induced involving key enzymes of the immune response. In the cv. ‘Royal Summer’, the genes linked to jasmonic acid/ethylene pathway were primarily upregulated. The genes JA and ETR1 are involved in the early step of the ethylene signal transduction pathway [79], ACC is responsible for the final step in ethylene biosynthesis, and PG plays a major role in both plant defense and controlling senescence [80]. Interestingly, these genes reached peak expression mainly at 10 hpi along with some effector genes (e.g., NEPs, Egh16, Rnt2, UkE1, and UkE2 genes). In the cv. ‘Royal Summer’, the SA was also upregulated mainly at 10 hpi, but contrary to what might be expected, PR-1 protein used to monitor SA and SAR activation [81] was downregulated at 10 hpi. Recent progress has revealed that plant PR-1 can activate an immune response. However, the activated pathway signaling can be blocked by pathogenic effectors to evade plant defense [82]. In addition, metabolome and transcriptome investigations on peach after *M. fructicola* inoculation indicated that the first peach response to infection occurred through the jasmonic acid signal molecule at 12 hpi, while the salicylic acid signal molecules were upregulated only after 48 hpi, emphasizing the plant response modulation to the pathogen [67]. In the ‘Messapia’ cv., both SA and PR1 were stimulated while the main genes linked to jasmonic acid, ethylene, and senescence (e.g., JA, ACC, and PG) were downregulated with maximum peak at 10 hpi and at which time the ETR1 was also downregulated. These results demonstrate that peach cultivars in the early stages of pathogen invasion can activate a different defense response. However, synchronization over the time of the host plant responses to pathogens can be suggested in both cultivars. Then, coordinated regulation of host gene expression could occur during fruit infection. Hence, we speculate that exactly at 10 hpi, some effector genes show more homogeneousness expression among the isolates because some effector genes upregulated (e.g., NEPs, Egh16, Rnt2, and UkE3 genes) or downregulated (e.g., PG6) mainly at 10 hpi in the ‘Royal Summer’ cv., while in the ‘Messapia’ cv., this trend was not detected.

Although transcriptome investigation is necessary to clarify the involvement of specific metabolic pathways, our work demonstrates that different peach cultivars can inversely modulate key genes associated with ethylene production upon *M. fructicola* infection. Notably, previous work suggests that *Monilinia* spp. can alter ethylene biosynthesis over time to benefit from the consequences of an ethylene burst likely on cell wall softening [83]. However, the same authors found the modulation of ethylene production linked to different *Monilinia* strains and stage of peach development.

## 5. Conclusions

In this work, we validated the involvement of putative effectors previously identified in the *M. fructicola* genome by assessing their expression in the host. We found that all the studied genes are involved, and despite the differences observed among the fungal isolates, they are modulated in relation to the host. Although the complexity of the host–pathogen interaction requires a holistic view of all associated proteins, our work indicates that in the battle between plant and pathogen, the host plant strategies against the pathogens are modulated over time. The plant–pathogen interaction mechanisms follow known models but involve different plant–pathogen molecular dialogue including molecules and different strategies in relation to peculiar characteristics of both the pathogen and the host. Therefore, the role of the host in eliciting the pathogen response should not be underestimated. In plants susceptible to brown root disease, we suggest the adaptation of the pathogen, which modulates its effector weapons based on the host’s physiology. This indicates that biotechnological genetic improvement programs will need to expand the number of target genes to effectively combat brown rot.

## Figures and Tables

**Table 3:** Ranking of reference genes according to their expression stability by geNorm. Means and standard deviations were determined with data from two biological replicates with three technical replicates each (n = 6).

	Target	Coefficient Variance	M-Value	Rank
*M. fructicola*	elongation factor 1-alpha	0.3606 ± 0.084	0.6894 ± 0.092	3
	β-tubulin	0.2381 ± 0.071	0.4357 ± 0.046	2
	β-actin	0.1732 ± 0.048	0.3661 ± 0.043	1
Peach	EF-1 elongation factor 1-alpha	0.3975 ± 0.097	1.0423 ± 0.072	4
	tubulin beta-1 chain	0.3194 ± 0.069	0.6445 ± 0.087	3
	β-actin	0.1512 ± 0.032	0.4131 ± 0.028	2
	histone H1	0.1234 ± 0.056	0.3647 ± 0.032	1

## Data Availability

The data supporting this study’s findings are available from the author, Lucia Landi, upon reasonable request.

## Supplementary Materials

The following supporting information can be downloaded at: https://www.mdpi.com/article/10.3390/jof11010039/s1, Table S1: Primers used in this study.
