# Supplementary material for: Validation of Monilinia fructicola Putative Effector Genes in Different Host Peach (Prunus persica) Cultivars and Defense Response Investigation
Source: J Fungi (Basel). 2025 Jan 6;11(1):39. doi: 10.3390/jof11010039 (PMC11766245; doi:10.3390/jof11010039)
Supplement: Supplementary file 1 [file jof-11-00039-s001.zip › jof-3381264-supplementary.pdf]

Supplementary Table S1: Primer pairs selected for gene expression analysis and related PCR amplification efficiencies data.

Locus_tag	Gene code	Primer Forward 5'-3'	Primer Reverse 5'-3'	Amp. size (bp)	Melt curve peak (°C)	Eff. (%)
EYC84_006718	NEP1	GCGGTGGATGGTATGGAAAC	TGGTGGGTTGGTGTTCTTCT	209	87.5	97.3
EYC84_009186	NEP2	TCTCAAGGTCGTCAATGGCT	AGGAGTACATGACGGCGTAG	170	85.5	99.4
EYC84_007944	Egh16	TCTTCAACAGCAAGAACGCC	TGGCTTGAGTACCTTGGGAG	234	85.5	92.2
EYC84_002620	CVNH	AGTCGAAGTCAGCGATGCTC	TGGAATCTCGAGCGAACAGC	196	85	94.3
EYC84_008853	PG6	GCAGCCCATAATTCAGACGG	AGACCATGACCTCCAAAGCA	152	83	104.2
EYC84_010609	PME	CTCAGCAAAACCACCACCTC	TGACGGTTCCTGTTTCCTCA	208	84.5	102.3
EYC84_008964	GAS1	GGTGTCGCATACCAGCAAGA	CAGAAGGAGTGGTCCCAACG	171	86	101.7
EYC84_001420	HsbA	TAACCCGCACCCTCAAATCC	AACCCGTCCGCAATTTTTGG	262	84.5	107.6
EYC84_008014	SSP	AAACCTTCGCCGTCTTCGAG	GCGACCGTAAACAAAGGTGC	245	87.5	95.7
EYC84_003936	GELP	TCCTTCTTGCCGGTGATTCT	TTCTTGGCGGGCTTTTGATC	250	87	98.7
EYC84_000899	TLP	TTGGTACTCAAGCGGGAACT	GCACCCGATCCATCATTTCC	178	84	96.5
EYC84_005201	Rnt2	TACCTCCCTACACCGGTACA	AGAAATAGGAGAGGGCGGTG	276	86	92.4
EYC84_002145	ycaC	GGACCAAACAACGCACTTCC	ATCGCTGAGAGTACCGCTTG	249	85	95.1
EYC84_004547	RPC5	GAAGTTCTCAGTCGCAACCC	TCTCTTTTCTTCGACGCAGC	180	85.5	97.6
EYC84_003395	UkE1	ATGGTCAAATTCTCCGCCCT	TTTGCACAGATAGAGGGGCA	152	85.5	97.3
EYC84_005228	UkE2	AGGCCTTCTGACCAAGTTCG	ACCGTTTGCCTTGTTGAAGC	259	83.5	101.1
EYC84_011230	UkE3	TAGCTTCTTTGGTCCCGAGC	GTTCTCTTTGCATGCCGGAC	208	85	102.7
**EYC84_004375**	ACTIN	CGACAATGCGATCCACAACC	CGACTACTAATGCGGTCGCT	389	87	108.3
**EYC84_002797**	TUBULI N	TTGCTTCGCAACTTGTGTCG	CCGTATATTGCCAGCGGGAT	392	84	103.4
**EYC84_000787**	elongati on factor alfa	GTGCGGAGGAATTGACAAGC	CAGCGGCAATGATGAGAACG	266	85.5	98.7
NM_001405051.1	PG	GCAGGAACGTTCTTTCTTCG	ACAACTCTCGCCATGAGAGG	244	82.5	99.5
AF124527.1	ETR-1	GATCGAAGGAGATGCTTTGG	GCCTTGACATGCTCTTCCTT	215	82	102.4
XM_007203058.2	SA	ATTCGTTGTGTCTGGGCAAG	TCAGCAATGTTTTGGAGGTG	198	82	106.7
JF694923.1	PR-1	GATATGCAAACTCCCGTGCT	CAAACCACCTGCGTGTAATG	195	85	93.4
XM_020560994.1	JA	CCGACATTGGCTTCAAAAAT	GATCCACATTCACCCCAATC	210	82.5	98.7
XM_007215636.2	ACC	AACTTTGGCCACACCCATAA	AAGCTCTATGCTGGCCTCAA	221	83	95.2
**XM_007215891.2**	GST	TCCATGCATGTGAGGAGAAA	TGCCCCAATTTAGCTTCATC	185	82	97.1
**XM_007205304.2**	β actin	GGAGCGTGGTTATTCCTTCA	TGCAGATTCCATTCCAATCA	232	84.5	96.2
**XM_007217941.2**	tubulin beta-1 chain	GGTTGGGTTTGCTCCACTTA	TCATCTTGCCACGGAACATA	199	85	102.1
**XM_034347152.1**	Histone H4	AGGAGGCGTGAAGAGAATCA	TTGAGCGCGTAGACAACATC	214	84.5	93.7
**XR_002271124.1**	**elongati on factor 2**	CCCTTACCGACTCCCTTGTT	TGAAAAGTCAACGTGCCCAG	236	85	95.7

Note: Amp = Amplicon; Eff= PCR efficiency; Bold= reference genes
